# Relation of Epicardial Fat Thickness with Carotid Intima-Media Thickness in Patients with Type 2 Diabetes Mellitus

**DOI:** 10.1155/2013/769175

**Published:** 2013-05-09

**Authors:** Mustafa Cetin, Musa Cakici, Mustafa Polat, Arif Suner, Cemil Zencir, Idris Ardic

**Affiliations:** ^1^Department of Cardiology, School of Medicine, Adiyaman University, 02000 Adiyaman, Turkey; ^2^Adiyaman University Research Hospital, Kahta Street, 02000 Adiyaman, Turkey; ^3^Department of Cardiology, Kahramanmaras State Hospital, 46000 Kahramanmaras, Turkey; ^4^Department of Cardiology, School of Medicine, Kahramanmaras Sutcu Imam University, 46000 Kahramanmaras, Turkey

## Abstract

*Aims.* The aim of this study was to investigate the relationship of echocardiographic epicardial fat thickness (EFT) with carotid intima-media thickness (CIMT), in patients with type 2 diabetes mellitus (T2DM). *Methods and Results.* A total of 139 patients with T2DM (mean age 54.3 ± 9.2 and 49.6% male) and 40 age and sex-matched control subjects were evaluated. Echocardiographic EFT and ultrasonographic CIMT were measured in all subjects. Patients with T2DM had significantly increased EFT and CIMT than those of the controls (6.0 ± 1.5 mm versus 4.42 ± 1.0 mm, *P* < 0.001 and 0.76 ± 0.17 mm versus 0.57 ± 0.14 mm, *P* < 0.001, resp.). EFT was correlated with CIMT, waist circumference, BMI, age, duration of T2DM, HbA1c in the type 2 diabetic patients. Linear regression analysis showed that CIMT (*β* = 3.52, *t* = 3.72, *P* < 0.001) and waist circumference (*β* = 0.36, *t* = 2.26, *P* = 0.03) were found to be independent predictors of EFT. A cutoff high risk EFT value of 6.3 mm showed a sensitivity and specificity of 72.5% and 71.7%, respectively, for the prediction of subclinical atherosclerosis. *Conclusion.* We found that echocardiographic EFT was significantly higher in patients with T2DM. Our study also showed that EFT was strongly correlated with waist circumference and CIMT as being independent of sex.

## 1. Introduction

Type 2 diabetes mellitus (T2DM) is one of the most common chronic diseases in the worldwide, the incidence of which tends to grow steadily. It is associated with a high risk of cardiovascular disease (CVD) which is the leading cause of death in patients with type 2 diabetes mellitus [1].

Obesity, insulin resistance, and diabetes have identified a proinflammatory state associated with increased adiposity [2]. Epicardial adipose tissue (EAT) is a visceral fat depot of the heart located along the large coronary arteries and on the surface of the ventricles and apex [3]. The embryological origin of EAT is similar to intra-abdominal visceral adipose tissue [4]. Several studies have shown that EAT is not only an anatomic depot of fat but also may serve as a local source of proinflammatory cytokines related to coronary artery disease (CAD) [5]. Therefore, EAT thickness has been considered to be a possible cardiovascular risk indicator [6, 7]. Transthoracic echocardiography (TTE), magnetic resonance imaging (MRI), and multislice computed tomography (MSCT) scanning have been conventional methods for quantifying EAT [8]. Assessment of EAT by TTE could be a simple and practical tool for cardiovascular risk stratification in clinical practice [3].

Carotid intima-media thickness (CIMT) is a simple and inexpensive tool to assess the cumulative effect of atherosclerotic risk factors and is an independent predictor of future cardiovascular (CV) risk [9]. The ultrasound-based measurement of CIMT has become a standard for assessing arteriosclerosis and is recommended by the American Heart Association for the noninvasive assessment of cardiovascular risk [10, 11].

Previous studies have reported that increased EAT is associated with CAD, metabolic syndrome (MetS) and obesity [12–16]. In the present study, we evaluated type 2 diabetic patients to investigate epicardial fat thickness by TTE and investigate its relationship with CIMT.

## 2. Methods

### 2.1. Patient Population

In this observational, cross-sectional study, 139 type 2 diabetic patients, having this diagnosis for at least 1 year, were consecutively included in the study. The control group consisted of 40 sex and age-matched healthy people. T2DM was diagnosed according to the American Diabetes Association criteria [17]. The study protocol was approved by our local ethics committee, and all patients gave their written informed consent to participate in the study. 

Exclusion criteria of the study were subjects with known ischemic heart disease, cerebrovascular disease, peripheral vascular disease, congestive heart failure, valvular heart disease, and chronic kidney disease.

 Medical history was obtained and physical examination was performed in all patients and controls. Blood pressure was measured three times—5 min apart—in a sitting position, on the right arm, and the mean value was calculated. Weight and height of the patients were measured without heavy outer garments and shoes, after a 12 h fasting period. Body-mass index (BMI) was calculated as body weight divided by the square of the height. Waist circumference was measured at the level of midway between the lower rib margin and iliac crest after removal of the clothes. Blood samples were withdrawn by venipuncture from all subjects following 12 h of fasting. Fasting blood glucose, serum creatinine, total cholesterol, high-density lipoprotein cholesterol (HDL), low-density lipoprotein cholesterol (LDL), and triglyceride levels were recorded. Glucose, creatinine, and lipid profile were determined using standard methods. Hemoglobin A1c (HbA1c) levels were measured by high pressure liquid chromatography with a thermo system. Serum CRP levels were evaluated using the nephelometric method.

### 2.2. Measurements of Epicardial Adipose Tissue Thickness

Each patient underwent a complete transthoracic echocardiography using the American Society of Echocardiography guidelines of measurement [18]. Echocardiogram was performed using a Vivid 7 (General Electronic, Wauke-sha, Wisconsin, USA) with a 2.5–3.5 MHz transducer, placed on the III–IV left intercostal space along the parasternal line, with patients being supine in left lateral decubitus and the head of the bed kept at 30°. All examinations were performed by an experienced cardiologist, blind to the patient's clinical information. Epicardial fat was identified as the space or layer anterior to the right ventricle with decreased echoreflectivity compared with the myocardium and pericardium. Epicardial fat thickness (EFT) was measured in end diastole on the free wall of the right ventricle from the parasternal long- and short-axis views, as previously described [19, 20]. The maximum values at any site were measured, and the average value was considered. Imaging constraints were used to ensure that the epicardial fat thickness was not measured obliquely. The intraobserver correlation coefficient was 0.96. Parasternal long- and short-axis views allow the most accurate measurement of EAT on the right ventricle, with optimal cursor beam orientation in each view.

### 2.3. Carotid Ultrasonography

Carotid arteries were evaluated using a Logiq 7 (General Electronic, Wauke-sha, Wisconsin, USA) with a 7.5 MHz transducer. All examinations were performed by an experienced radiologist, blind to the patient's clinical information. Measurements involved a primary transverse and longitudinal scanning of the common carotid artery, bifurcation, and internal carotid. The CIMT was measured on the far wall at 1 cm from bifurcation of the common carotid artery as the distance between the lumen-intima interface and the media-adventitia interface. At least three measurements were performed on both sides, and the average measurement was taken as the CIMT. All measurements were made at a plaque-free site. The intraobserver correlation coefficient was 0.97. 

### 2.4. Statistical Analysis

SPSS statistical software (SPSS for Windows, version 17.0, Inc., Chicago, IL, USA) was used for all statistical calculations. Categorical variables were expressed as number and proportions, while continuous variables were expressed as mean ± standard deviation. Chi-square (*χ* ^2^) test was used to compare groups regarding categorical variables. Continuous variables were compared with Student *t*-test (while comparing parametric variables between diabetic patients and controls) or Mann-Whitney *U* test (while comparing nonparametric variables between diabetic patients and controls). Correlation analysis was performed using Pearson or Spearman tests. Linear regression analysis was used to explore the independent determinants of EFT. Receiver operating characteristic (ROC) curve analysis was performed to determine cutoff high risk value of EFT when patients are divided into two groups according to CIMT (< or ≥ 0.9 mm). Levels of statistical significance were set at a *P* value <0.05.

## 3. Results

There were 139 patients (mean age 54.3 ± 9.2 and 49.6% male) in the T2DM group and 40 patients (mean age 52.1 ± 7.3 and 50% male) in the control group. The demographic findings and laboratory values of the study groups are presented on Table 1. The mean duration of disease in patients with T2DM was 6.5 ± 3.9 years. The Age, frequencies of sex distribution, hypertension, hyperlipidaemia, smoking, family history of the CAD, and waist circumference were similar between patients with T2DM and the controls. BMI was higher in the control group compared with the value of the type 2 diabetic patients (29.1 ± 4.2 versus 27.6 ± 3.1, *P* = 0.03, resp.). Patients with T2DM had significantly increased EFT and CIMT than those of the controls (6.0 ± 1.5 mm versus 4.42 ± 1.0 mm, *P* < 0.001 and 0.76 ± 0.17 mm versus 0.57 ± 0.14 mm, *P* < 0.001, resp.). When laboratory findings were compared, type 2 diabetic patients had significantly higher fasting blood glucose, creatinine, C-reactive protein levels, and HbA1c, but total cholesterol, LDL, HDL and triglyceride levels did not differ between the two groups.

**Figure 1:**
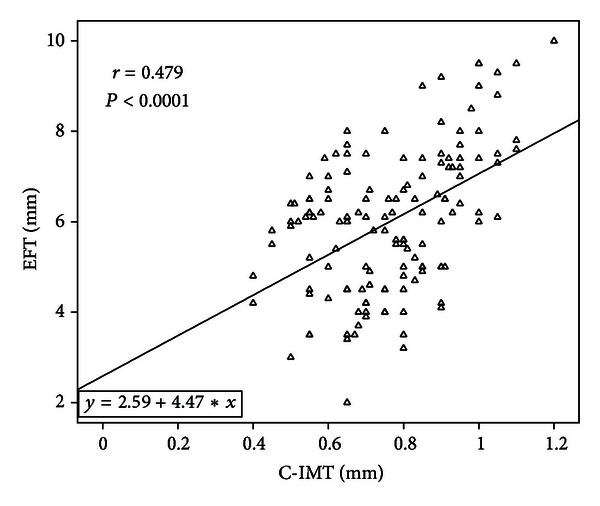
Epicardial fat thickness (EFT) is positively correlated with carotid intima-media thickness (CIMT) in diabetic patients.

**Figure 2:**
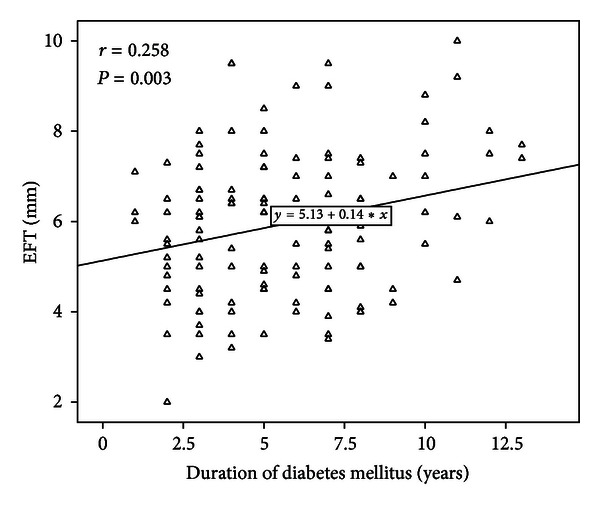
Duration of diabetes mellitus is linear and positively correlated with epicardial fat thickness (EFT).

The variables correlated with EFT in the type 2 diabetic patients were CIMT (*r* = 0.479, *P* < 0.001) (Figure 1), waist circumference (*r* = 0.371, *P* < 0.001), BMI (*r* = 0.315, *P* < 0.001), age (*r* = 0.260, *P* = 0.002), duration of DM (*r* = 0.258, *P* = 0.003) (Figure 2), and HbA1c (*r* = 0.200, *P* = 0.032) (Table 2), and those in the controls were; CIMT (*r* = 0.690, *P* < 0.001), waist circumference (*r* = 0.420, *P* = 0.02), and age (*r* = 0.365, *P* = 0.02). When we performed subgroup analysis in the patients with T2DM according to sex, EFT was correlated with CIMT (*r* = 0.481, *P* < 0.001), waist circumference (*r* = 0.429, *P* < 0.001), age (0.348, *P* = 0.003), BMI (*r* = 0.391, *P* < 0.001), duration of DM (*r* = 0.293, *P* = 0.014), and weight (*r* = 0.285, *P* = 0.018) in female patients and CIMT (*r* = 0.481, *P* < 0.001) and waist circumference (*r* = 0.263, *P* < 0.03) in male patients. After multivariate stepwise linear regression analysis, CIMT (*β* = 3.52, *t* = 3.72, *P* < 0.001) and waist circumference (*β* = 0.36, *t* = 2.26, *P* = 0.03) were found to be independent relevant factors of EFT in patients with T2DM (Table 3). 

**Figure 3:**
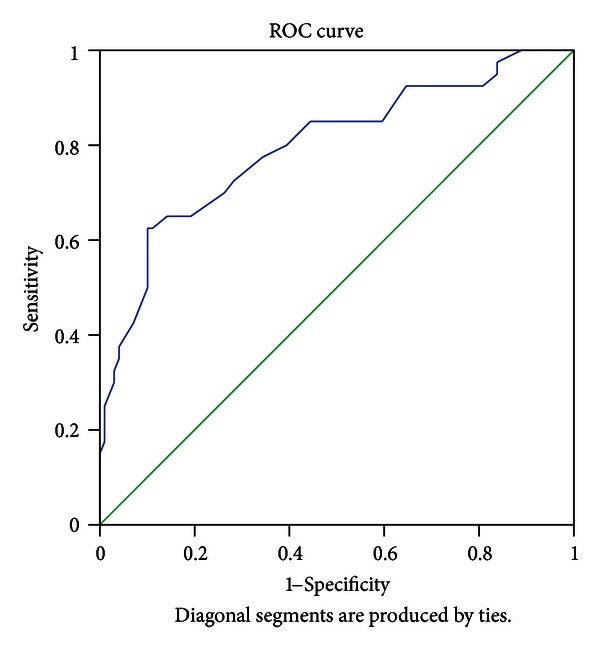
Receiver operating characteristics (ROC) curve (showing sensitivity and specify of the epicardial fat thickness to determine cutoff high risk value). The area under the ROC curve of 0.797 indicates a very good accuracy of the test.

The patients with T2DM are divided into two groups according to the values of CIMT (< or ≥0.9 mm), which is an indicator level of subclinical organ damage as proposed by ESC hypertension guideline [21], to determine cutoff high risk value of EFT in patients T2DM by receiver operating characteristic (ROC) curve analysis. In the ROC curve analysis, the area under the curve (AUC, Figure 3) was found statistically significant (AUC = 0.797, 95% CI: 0.709–0.884, *P* < 0.001). As an optimal cutoff point, high risk EFT value of 6.3 mm was determined with a 72.5% sensitivity and 71.7% specificity.

## 4. Discussion

The major findings of the present study were first, patients with T2DM had increased EFT and CIMT compared with age- and sex-matched controls. Second, EFT was correlated with CIMT, waist circumference, BMI, age, duration of DM, and HbA1c in patients with T2DM. Third, CIMT and waist circumference were found to be independent predictors of EFT. Finally, EFT of 6.3 mm was determined as a high risk value for subclinical atherosclerosis with a 72.5% sensitivity and 71.7% specificity in ROC curve analysis.

 Epicardial, mesenteric, and omental fats share the same origin from the splanchnopleuric mesoderm [4]. It has been shown that EAT produces inflammatory mediators such as interleukin (IL)-6, IL-1b, tumor necrosis factor (TNF)-a, and monocyte chemotactic protein (MCP-1) in patients with significant coronary artery disease [22] and expresses mRNAs of adiponectin, resistin, leptin, IL-6, TNF-a, and CD-45 [23]. Several studies have shown that EAT can play a role in the development and aggravation of CAD [8, 22–24]. In addition, EFT has been shown to be related to MetS, abdominal visceral adiposity, subclinical atherosclerosis, nonalcoholic fatty liver disease, type 1 DM, impaired fasting glucose, and hypertension [19, 20, 25–29]. 

To our knowledge, as a main difference when compared to previously published data in patients with MetS, this is the first study in the literature focusing on the relationship between EFT and CIMT in patients with T2DM. It is also important that a positive linear and significant relationship between EFT and duration of diabetes mellitus was found in our study. According to these results, EFT may be used as a marker of subclinical atherosclerosis and disease progression in patients with T2DM. Further studies are required to support this hypothesis. 

There is very limited study investigating the relationship between T2DM and EFT. Recently, in a study performed by Kim et al. [30], increased EAT thickness assessed by cardiovascular magnetic resonance (CMR) was an independent risk factor for significant coronary artery stenosis in asymptomatic type 2 diabetes. There were 100 patients and no control group in that trial. In another study reported in a series of 49 type 2 diabetic patients by Wang et al. [31], EAT volume assessed by cardiac multislice computed tomography was shown to be increased and was associated with unfavourable components of MetS and coronary atherosclerosis. In our study, similar to these trials, we found that EFT was increased in patients with T2DM. In contrast to these trials, we evaluated EFT by TTE in a larger population with a control group and sought relation between EFT and CIMT, which is increasingly used as a surrogate marker for atherosclerosis. Although epicardial fat is readily visualized on high-speed CT and MRI, widespread use of these methods for its assessment is not practical. Echocardiographic EFT measurement in the current practice appears to be feasible, as well as reliable due to good reproducibility which has been shown both in previous studies [19, 20, 32] and in this study.

In our study, patients with T2DM had lower BMI. However, EFT and CIMT were higher in the diabetic patients compared to the controls. Additionally, BMI correlated with EFT in female diabetic patients, but it did not correlate with EFT in male diabetic patients and did not find independent predictor for EFT. Waist circumference not only was correlated with EFT but also was independent predictor for EFT in patients with T2DM and also in both sex. It has been reported that BMI is not a good measure of body adiposity [33]. Similarly, we also found that waist circumference was more reliable parameter than BMI to predict EFT in this study. 

Iacobellis et al. [34] have reported that threshold values of high risk EFT to predict MetS are median values of 9.5 mm (85% sensitivity and 63% specificity) and 7.5 mm (82% sensitivity and 62% specificity) in white men and women, respectively. In another study performed by Natale et al. [35] in a large population of hypertensives, Patients with EFT > 7 mm showed a significantly increase in CIMT (0.84 ± 0.2 mm). In a large study performed on patients presenting for cardiovascular preventive care, Nelson et al. [36] have reported that EAT thickness ≥5.0 mm may identify an individual with a higher likelihood of having detectable carotid atherosclerosis. In our study, we found that EFT of 6.3 mm was determined as a high risk value for subclinical atherosclerosis with a 72.5% sensitivity and 71.7% specificity. Different race and patient population in those studies may have created these different threshold values of EFT. Even if different threshold values of EFT have been found in all these studies, increased EFT seems to be related to atherosclerotic process. 

Our study had some limitations. This is a case control study, and prospective studies are necessary to show relation between EFT with CIMT and waist circumference. All data were based on a single measurement and may not reflect the association of EFT and CIMT regarding changes with time. In order to establish EFT as a high risk criteria of atherosclerosis in type 2 diabetic patients, further studies are necessary on larger series.

In conclusion, we found that EFT measure by TTE was increased in patients with T2DM. Our study also demonstrated that EFT was strongly correlated with waist circumference and CIMT as being independent of sex. Threshold value of high risk EFT was determined to be 6.3 mm. These results from our study population may suggest that the echocardiographic assessment of EFT is the reliable marker of atherosclerosis and increased CV risk in patients with T2DM. Further studies are needed to confirm these findings.

## Figures and Tables

**Table 1:** Clinical and biochemical characteristics of the patients with type 2 diabetic patients and healthy controls.

Parameters	DM (*n* = 139)	Controls (*n* = 40)	*P* value
Age (years)	54.3 ± 9.2	52.1 ± 7.3	0.109
Gender (male, %)	49.6%	50%	0.968
Disease duration (yrs)	6.5 ± 3.9	—	—
Hypertension (%)	41%	35%	0.494
Smoking (%)	20.9%	15%	0.410
Hyperlipidaemia (%)	35.3%	32.5%	0.747
Family history of CAD (%)	16.5%	15%	0.815
Epicardial fat thickness (mm)	6.0 ± 1.5	4.42 ± 1.0	<0.001
Carotid artery intima-media thickness (mm)	0.76 ± 0.17	0.57 ± 0.14	<0.001
Body mass index (kg/m^2^)	27.6 ± 3.1	29.1 ± 4.2	0.03
Waist circumference (cm)	90.8 ± 9.3	89.1 ± 11.2	0.392
Systolic blood pressure (mm Hg)	133.9 ± 17.6	129.4 ± 12.4	0.209
Diastolic blood pressure (mm Hg)*	78 (40–100)	80 (60–90)	0.440
Fasting glucose (mg/dL)	147 ± 38.8	94.1 ± 6.3	<0.001
Creatinine (mg/dL)	0.88 ± 0.15	0.77 ± 0.17	0.03
Total cholesterol (mg/dL)	199.1 ± 28.1	198.4 ± 23.9	0.933
LDL (mg/dL)	116.1 ± 25.0	118.3 ± 18.0	0.734
HDL (mg/dL)	43.5 ± 9.4	46.6 ± 10.0	0.247
Triglyceride (mg/dL)	172 ± 72.7	163.2 ± 85.2	0.681
C-reactive protein (mg/dL)	3.0 (2–13.1)	1.9 (0.93–7.6)	0.002
HbA1c (%)	7.4 ± 1.2	5.1 ± 0.41	<0.001
Hemoglobin (g/dL)	14.2 ± 1.6	14.3 ± 1.7	0.741
Platelets (×10^9^/L)	276.2 ± 70.4	251.5 ± 42.9	0.167

Data are presented as mean ± s.d. or *n* (%). CAD: coronary artery disease; HDL: high-density lipoprotein; LDL: low-density lipoprotein. *Diastolic blood pressure is presented as the median (min–max).

**Table 2:** The univariate correlations of the epicardial fat thickness.

Variable	*r*	*P*
Carotid artery intima-media thickness	0.479	<0.001
Waist circumference	0.371	<0.001
Body mass index	0.315	<0.001
Age	0.260	0.002
Disease duration	0.258	0.003
HbA1c	0.200	0.032
Weight	0.165	0.054

The correlations with a *P* > 0.10 are not shown.

**Table 3:** Independent predictors for epicardial fat thickness by multivariate stepwise linear regression analysis.

Model	Unstandardized coefficients		Standardized coefficients	*t*	*P* value	95.0% confidence interval for *B*	
	*B*	Std. error	Beta			Lower bound	Upper bound
Carotid artery intima-media thickness	3.52	0.946	0.362	3.719	<0.001	1.64	5.40
Waist circumference	0.36	0.016	0.220	2.263	0.026	0.004	0.068

The model including CIMT, age, weight, HbA1c, waist circumference, duration of DM and BMI. *B*: Coefficient of regression.
